# A dosimetric phantom study of dose accuracy and build-up effects using IMRT and RapidArc in stereotactic irradiation of lung tumours

**DOI:** 10.1186/1748-717X-7-79

**Published:** 2012-05-31

**Authors:** Jan Seppala, Sami Suilamo, Jarmo Kulmala, Pekka Mali, Heikki Minn

**Affiliations:** 1 Department of Oncology and Radiotherapy, Turku University Hospital, POB 52, 20521, Turku, Finland; 2 Cancer Center, Kuopio University Hospital, POB 1777, 70211, Kuopio, Finland; 3 Turku PET Centre, Turku University Hospital, POB 52, 20521, Turku, Finland

**Keywords:** Stereotactic body radiotherapy, Lung cancer, IMRT, Heterogeneity, Surface dose

## Abstract

**Background and purpose:**

Stereotactic lung radiotherapy (SLRT) has emerged as a curative treatment for medically inoperable patients with early-stage non-small cell lung cancer (NSCLC) and the use of intensity-modulated radiotherapy (IMRT) and volumetric modulated arc treatments (VMAT) have been proposed as the best practical approaches for the delivery of SLRT. However, a large number of narrow field shapes are needed in the dose delivery of intensity-modulated techniques and the probability of underdosing the tumour periphery increases as the effective field size is decreased. The purpose of this study was to evaluate small lung tumour doses irradiated by intensity-modulated techniques to understand the risk for dose calculation errors in precision radiotherapy such as SLRT.

**Materials and methods:**

The study was executed with two heterogeneous phantoms with targets of Ø1.5 and Ø4.0 cm. Dose distributions in the simulated tumours delivered by small sliding window apertures (SWAs), IMRT and RapidArc treatment plans were measured with radiochromic film. Calculation algorithms of pencil beam convolution (PBC) and anisotropic analytic algorithm (AAA) were used to calculate the corresponding dose distributions.

**Results:**

Peripheral doses of the tumours were decreased as SWA decreased, which was not modelled by the calculation algorithms. The smallest SWA studied was 2 mm, which reduced the 90% isodose line width by 4.2 mm with the Ø4.0 cm tumour as compared to open field irradiation. PBC was not able to predict the dose accurately as the gamma evaluation failed to meet the criteria of ±3%/±1 mm on average in 61% of the defined volume with the smaller tumour. With AAA the corresponding value was 16%. The dosimetric inaccuracy of AAA was within ±3% with the optimized treatment plans of IMRT and RapidArc. The exception was the clinical RapidArc plan with dose overestimation of 4%.

**Conclusions:**

Overall, the peripheral doses of the simulated lung tumours were decreased by decreasing the SWA. To achieve adequate surface dose coverage to small lung tumours with a difference less than 1 mm in the isodose line radius between the open and modulated field, a larger than 6 mm SWA should be used in the dose delivery of SLRT.

## Background and purpose

Stereotactic lung radiotherapy (SLRT) is an effective treatment option for malignant pulmonary tumours that measure 6 cm or less [1,2]. In SLRT a high dose of radiation is given with few treatment fractions and a high probability of tumour control can be achieved when compared to the conventional fractionation [3,4]. Clinical outcomes of SLRT for peripheral primary lung tumours are comparable to surgery and this is one of the reasons that SLRT is rapidly increasing in the treatment of small lung tumours [5,6]. The use of intensity-modulated radiotherapy (IMRT) and volumetric modulated arc treatments (VMAT) has been evaluated and proposed over 3D-CRT techniques for the delivery of SLRT [7-9]. However, with the increased complexity in beam shaping a large number of narrow field shapes are needed in the dose delivery. This situation becomes a clinical concern when the irradiated volume is located in lung tissue, where the secondary Compton electrons have a wider range. Dose rebuild-up and rebuild-down effects occur in the tumour periphery and the effects become steeper when the field size is decreased [10]. As a result underdosing effects take place at the lung-tumour interface potentially decreasing the minimum dose delivered to the tumour, which in turn can decrease the tumour control probability [11].

The inadequacy of the most common, type-a algorithms, such as pencil beam convolution (PBC) to calculate the dose accurately inside heterogeneous media is well documented [12,13]. The type-b calculation algorithms, such as collapsed cone (CC) and anisotropic analytic algorithms (AAA) are able to approximate the electron transport in heterogeneous media more accurately than the PBC algorithm [14-16]. The dosimetric accuracy of these algorithms is, however, controversial and depends on field size, beam energy and density of lung investigated. Monte Carlo simulation is assumed to be the best representation of the real dose distribution. Recently new Monte Carlo codes have been developed, which allow the simulations of complex IMRT and VMAT delivery techniques [17]. Unfortunately, full Monte Carlo have not gained widespread clinical use yet since the simulations with high spatial resolution are laborious and time-consuming.

The accuracy of different dose calculation algorithms has been studied in the treatments of SLRT mainly with the conventional 3D-techniques with relatively large field sizes as compared to the effective field sizes used in intensity modulated techniques [12,18,19]. Numerous studies have also compared the calculated doses of type-a and type-b algorithms but unfortunately the measurements with IMRT dose delivery methods have been sparse [20,21]. To our knowledge, dose accuracy of IMRT or RapidArc, a form of VMAT, in a heterogeneous phantom has been performed only once [22]. However, studies to investigate the effect of small MLC apertures of the IMRT or RapidArc to the surface doses of small tumours have not been conducted. The aim of this work was to evaluate the dosimetric accuracy of IMRT and RapidArc techniques in SLRT using heterogeneous phantoms. Another purpose of this study was to define the smallest sliding window aperture (SWA) that can be used in SLRT without compromising the peripheral doses of the treated lung tumours.

## Methods and materials

The effect of lung heterogeneities on tumour central and peripheral doses was studied with a Novalis Tx linear accelerator (Varian Medical Systems, Palo Alto, CA and BrainLAB, München, Germany) with a photon energy of 6 MV. Higher energies were neglected since the dosimetric advantages of lower photon energies have been well documented in SLRT [15,23]. The Novalis Tx is equipped with a high-definition multileaf collimator (HD-MLC) with a total of 60 leaf pairs with central leaves of width 2.5 mm and the peripheral leaves 5 mm in the isocentre.

### Phantoms

**Figure 1:**
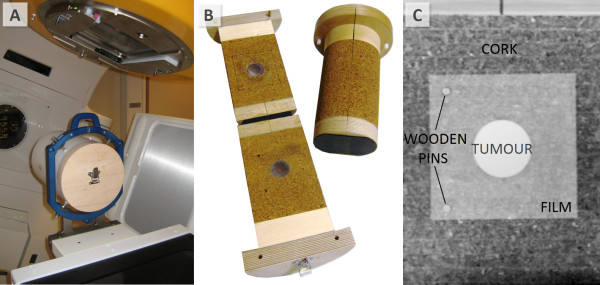
**Measurement set-up**. The outer PMMA cylinder was attached to a stereotactic frame (A) and the cork cylinders (B) were inserted inside. A frontal view of the Ø4.0 cm tumour surrounded by cork with film inside (C).

Two heterogeneous phantoms, made of polymethyl methacrylate (PMMA, ρ = 1.18 g/cm^3^), polycarbonate and cork, were designed for this study (Figure 1). Spherical polycarbonate inserts of Ø1.5 cm and Ø4.0 cm mimicked the lung tumours with an average density of 50 HU. Tumours were surrounded by cylindrical cork (average density of −550 HU) with a diameter of 14 cm. The phantoms were cut in half so that an EBT2 film (Gafchromic; ISP, Wayne, NJ) could be placed in the middle of the tumour (Figure 1B). The cylinders were designed so that they were reproducibly insertable to a cylindrical outer ring mimicking thoracic wall with a thickness of 3 cm constructed from PMMA. In addition to be able to accurately repeat the film positioning in the phantoms, two wooden pins were attached into the cork with fixed distances (Figure 1C). The films had the corresponding holes to fit these pins.

Two additional phantoms made of solid PMMA were constructed to execute measurements in homogeneous media with two ionization chambers (Farmer NE 2571 and PTW Micro chamber). The solid PMMA inserts had tailored cavities in the middle of the phantom to fit the chambers inside.

### Treatment planning

The phantoms were imaged with a 16-slice CT scanner (GE LightSpeed; General Electric Medical Systems, Milwaukee, WI) with a slice thickness of 1 mm. The CT-images were transferred to the treatment planning system (Eclipse^TM^, version 8.909, Varian Medical Systems). Tumours were contoured to the 3D-images of the phantoms and a margin of 5 mm was added to the tumour resulting in a planning target volume (PTV).

The rebuild-up effects in the tumours were studied with SWAs of 2, 4, 6, 10 and 15 mm created in Shaper software (Varian Medical Systems). The sliding apertures had a constant velocity with a constant dose rate of 500 MU/min. First only one anterior field was used to calculate the doses from open fields and from the created SWAs to the phantoms. The isocentres of the treatment plans were set 1 cm off axis from the centre of the tumours to reduce the attenuation effect of the film itself [24]. The secondary collimators had fixed asymmetric field sizes of 2.5 × 2.5 cm^2^ and 5 × 5 cm^2^ with the small and the large tumour, respectively. The movement of the known apertures started from outside the secondary collimators. In addition ‘clinical’ nine field treatment plans with equally spaced gantry angles were created to study the dose distributions in the tumours. A total of nine treatment plans with each tumour insert were studied: five treatment plans with the various SWAs, a plan with open fields of 2.5 × 2.5 cm^2^ or 5 × 5 cm^2^ (3D-CRT plan), an IMRT treatment plan with dynamic delivery and two RapidArc plans. IMRT treatment plan optimization focused only on the tumour dose coverage and homogeneity. The minimum dose to the tumour volume was set to be greater than 0.985 Gy and the maximum dose less than 1.015 Gy. With the first RapidArc plan (RA1) dose optimization constraints were only used for tumour dose coverage and uniformity with minimum dose objective to tumour volume 0.99 Gy and maximum dose objective less than 1.015 Gy with one fraction. The second RapidArc plan (RA2) optimization included also organs at risks specified in the phantom volume. The delineated volumes simulated heart and spinal cord with maximum dose limits of 0.2 Gy and 0.3 Gy, respectively. The arc rotations were 360^o^ with a collimator rotation of 45^o^ with both RA1 and RA2.

The dose distributions were calculated with PBC with modified Batho power law and with AAA (version 8.908) with the smallest calculation grid sizes available (1.25 mm and 1.0 mm, respectively). A prescription dose of 1 Gy was chosen since the clinical doses of 12-20 Gy were out of the range of the EBT2 films.

### Dose delivery

The outer PMMA-cylinder was fixed to the treatment table with a stereotactic head ring (BrainLAB AB) (Figure 1A). Cork cylinders were inserted into the outer cylinder either parallel or perpendicular to vertical axis and the isocentres were localized with CBCT and ExacTrac 6D. Films were irradiated both perpendicular and parallel to the central beam axis. Ionization chamber measurements were performed only with the fixed anterior beam in a homogeneous cylinder with the various SWAs and open fields, respectively.

### Film dosimetry

Two holes were cut to the films with fixed distances from each other to match the pins in the phantoms. The film measurements were repeated three times and averaged together to reduce the variability of an individual film.

From each sheet of the film two additional pieces (5 × 5 cm^2^) were irradiated to a dose of 1 Gy and two were set as reference films of 0 Gy. The 1 Gy reference films were used to rescale the measurements to account for the possible variation in the sensitivity of each sheet of a film. The films were scanned at RGB-mode at 72 dpi with no corrections in the scanner always at the same position at the centre of the scanning bed of Epson V700 scanner (Seiko Epson Corporation, Nagano, Japan) 24 h after the irradiation [25]. Scanner output variations were recorded by a fixed reference film located above the irradiated films.

Scanned films were analyzed with OmniPro I’mRT software (version 1.7, IBA Dosimetry, Schwarzenbruck, Germany). Within the software the films were aligned with respect the two fixed holes in the film and were centred to the middle of the upper hole. Responses of the films to irradiation were measured in the red colour channel and the optical densities were converted to dose according to the calibration films. The standard deviation (SD) of three independent measurements of the central doses of the tumours was used to quantify the uncertainty of the measured dose. The peripheral doses of the tumours were analyzed by measuring the X- and Y-coordinate widths of 50%, 80%, 85%, 90% and 95% isodose lines.

## Results

The ionization chamber dose measurements were compared to the calculations and the results are shown in Table 1. The largest deviations (maximum 6.6%) were observed with the smallest SWAs in the smaller tumour. The difference between AAA and PBC in the homogeneous phantom was small. The film measurements were performed in heterogeneous phantom both parallel and perpendicular to the beam axis and the results from the direct anterior beam measurements are shown in Table 1. PBC overestimated the central doses of the tumours irradiated by open fields with a maximum dose difference of 6.0%. As a consequence the calculated doses of the small SWAs were closer to the measured values with PBC than with the AAA since the calculated doses of the small SWAs were on the contrary underestimated. The measurement error was approximated by a SD of the three discrete film measurements with an average SD of 1.6% (range 0.3% - 3.7%). The variation in the scanner output was recorded from the reference film and was at maximum 0.6% (average <0.1%).

**Table 1:** Dose differences (Measured – Calculated) in the centre of Ø1.5 cm and Ø4.0 cm tumours of the single anterior field irradiations and the standard deviations (SD) of the three discrete film measurements

		**Chamber measurements**		**Film measurements, single field irradiations**					
		*Homogeneous Phantom*		*Heterogeneous Phantom, Perpendicular*			*Heterogeneous Phantom, Parallel*		
		Measured - AAA	Measured - PBC	Measured - AAA	Measured - PBC	SD	Measured - AAA	Measured - PBC	SD
		(cGy)	(cGy)	(cGy)	(cGy)	(cGy)	(cGy)	(cGy)	(cGy)
**Ø1.5 cm**	10 × 10 cm^2^	−1,3	−0,8	−0,9	−5,1	1,5	−1,8	−6,4	0,9
	2.5 × 2.5 cm^2^	−0,5	0,7	−0,7	−6,0	1,8	0,3	−3,9	1,8
	SWA 2 mm	5,7	6,6	5,8	−0,3	3,7	3,1	−1,6	1,8
	SWA 4 mm	3,4	4,4	5,1	−0,9	2,0	3,9	−0,8	2,7
	SWA 6 mm	2,0	3,0	2,8	−3,1	2,1	3,6	−1,0	2,5
	SWA 10 mm	1,9	2,9	3,4	−2,5	1,1	3,1	−1,6	1,8
	SWA 15 mm	1,4	2,4	1,5	−4,2	0,5	2,7	−1,9	2,0
**Ø4.0 cm**	10 × 10 cm^2^	−1,3	−0,8	−0,7	−4,6	0,3	−0,6	−4,4	1,4
	5.0 × 5.0 cm^2^	−0,7	0,2	−0,1	−0,4	0,7	2,3	2,3	0,6
	SWA 2 mm	1,4	2,4	1,5	1,7	1,4	−1,6	−1,1	0,9
	SWA 4 mm	0,8	1,9	2,1	2,3	1,9	2,0	2,5	2,6
	SWA 6 mm	0,8	1,9	1,5	1,6	1,0	0,6	1,1	1,7
	SWA 10 mm	0,3	1,4	1,2	1,3	1,7	4,2	4,7	1,8
	SWA 15 mm	0,5	1,6	1,1	1,3	1,7	4,0	4,5	1,1

**Figure 2:**
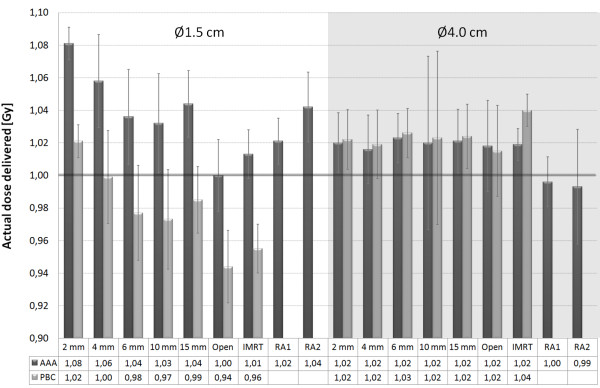
**Measured central doses of the tumours (Ø1.5 cm and Ø4.0 cm) with the calculated MUs of PBC and AAA**. The dose prescription was 1.0 Gy to the centre of the mimicked tumour. The error bars represent the 95% confidence intervals of the measurements.

**Figure 3:**
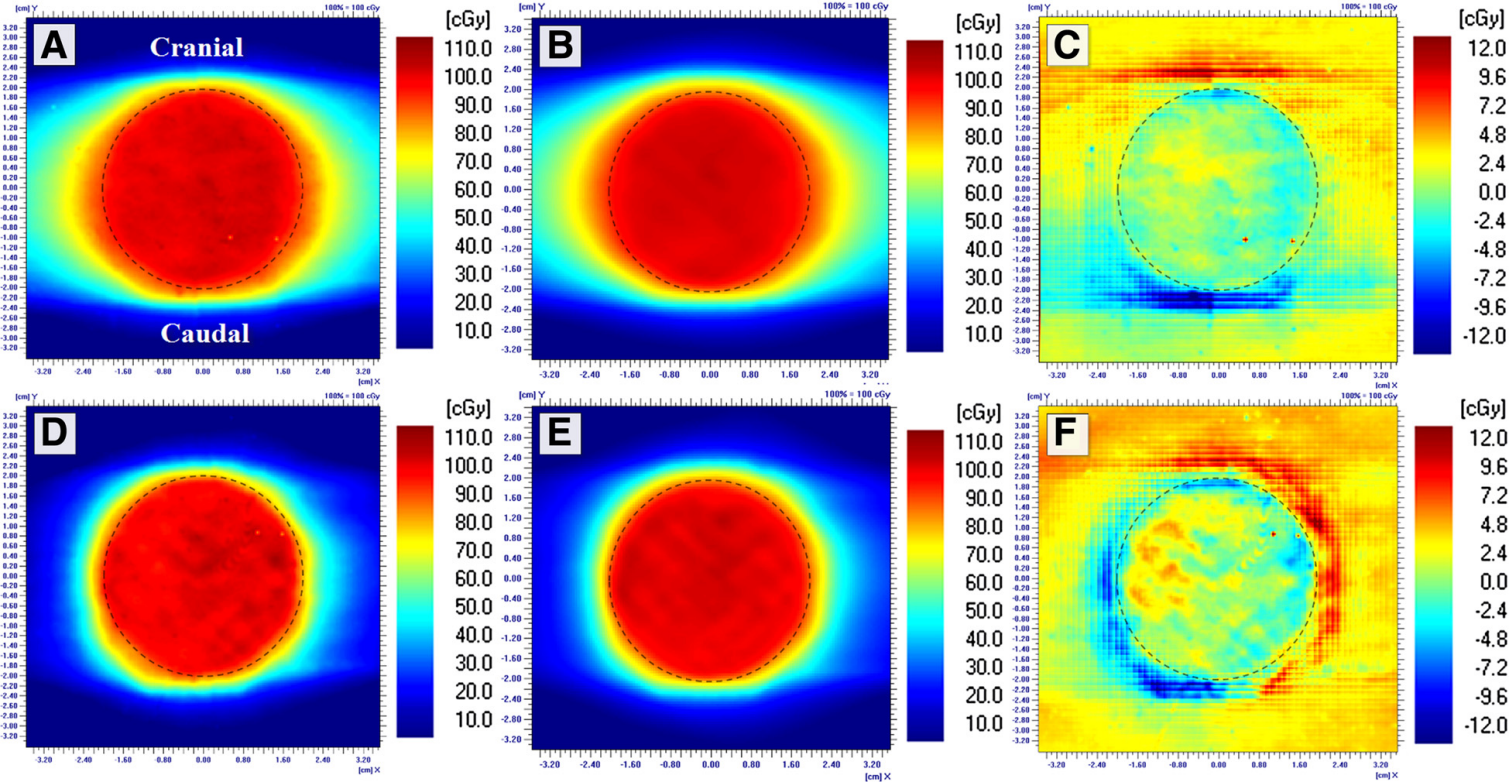
**Film measurements of the RA1 (A) and RA2 (D) treatment plans of the Ø4.0 cm tumour**. The corresponding dose distributions of AAA calculations (B and E) and the related absolute differences (C and F) (calculated - measured). The border of the tumour is visualized with a dashed line.

Dose in the centre of the simulated lung tumours was measured with various treatment plans. The actual doses delivered by the calculated monitor units (MU) by PBC and AAA are shown in Figure 2. The results were congruent with the single anterior field measurements as the differences between calculated and measured doses were greater with the smaller than with the larger tumour. Measurements of the RapidArc treatment plans deviated on average 2.7% (SD 1.9%) from the 3D-CRT plans, while with IMRT treatment plans the difference was on average 0.6% (SD 1.1%). With RA2 treatment plan the difference between planned and measured dose distribution was increased, as can be seen in Figure 3.

**Figure 4:**
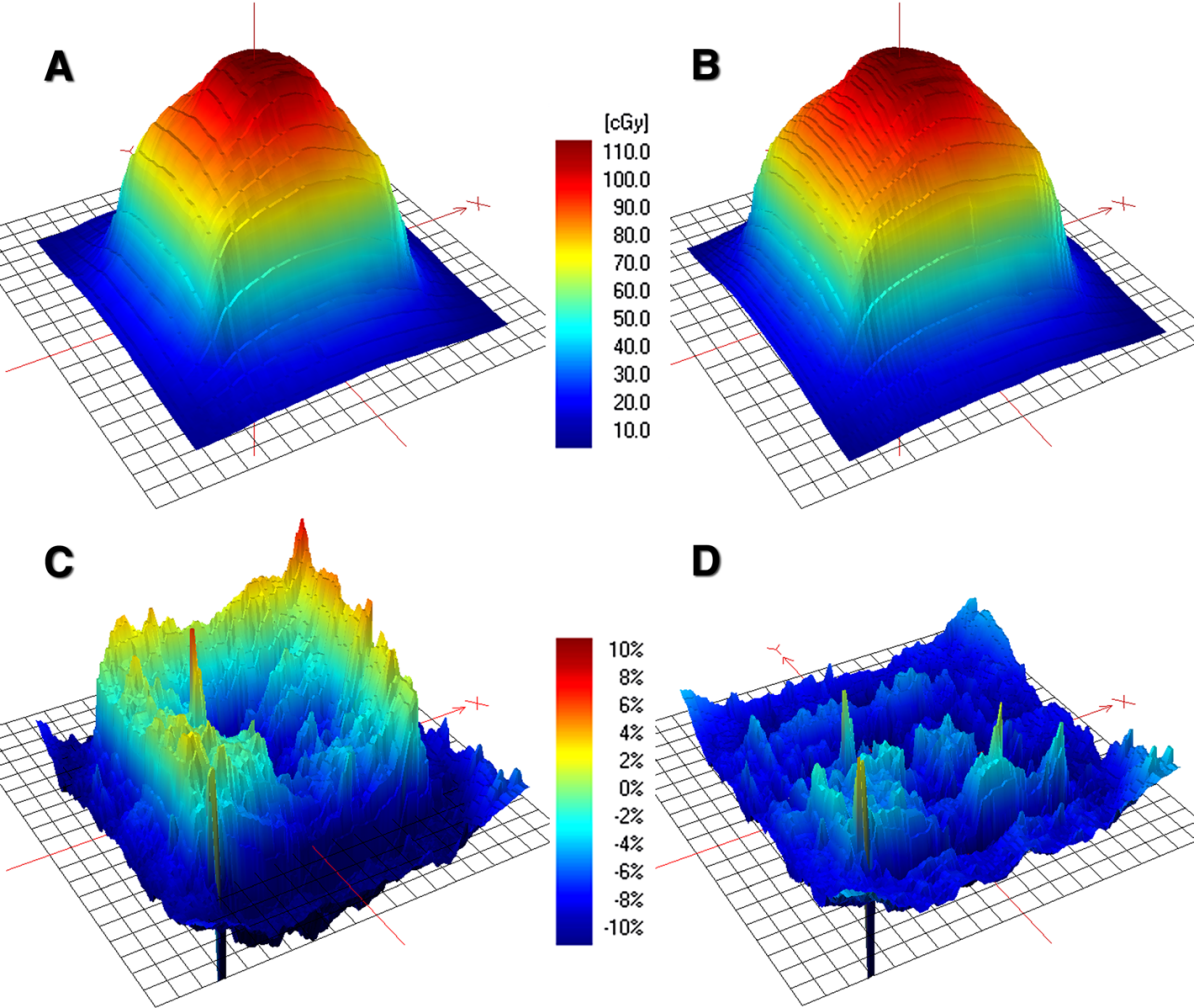
**Measured dose distributions of 2 mm (A) and 15 mm (B) SWAs were subtracted from the corresponding open field measurement of 2.5 × 2.5 cm**^**2**^**(C and D)**. Films were perpendicular to the beam axis in the middle of the Ø1.5 cm tumour.

**Figure 5:**
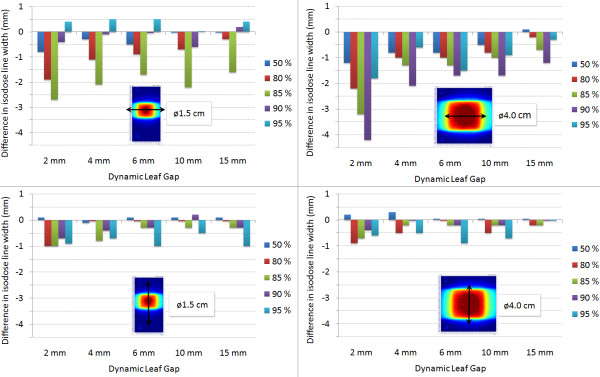
**Difference in measured surface doses represented by the difference in isodose line widths of the treatment plans of 3D-CRT and various SWAs (SWA - 3D-CRT)**. The mimicked lung tumours of Ø1.5 cm and Ø4.0 cm were irradiated with the nine field treatment plans. The widths of normalized isodose lines (50%, 80%, 85%, 90% and 95%) were measured from the irradiated films to X- and Y-direction.

Isodose line widths of 50%, 80%, 85%, 90% and 95% were studied to quantify the difference in surface doses from various SWAs. The film measurements from the open field irradiations (3D-CRT) were set as a reference width. With this we wanted to quantify the impact of the modulated fields to the surface doses of the tumours. The dose in tumour periphery was decreased as the SWA decreased, which is visible in Figure 4. With focus on the treatment plans the difference in isodose line width in the X- and Y-direction as a function of SWA is shown in Figure 5 (SWA – 3D-CRT). The X- and Y-directions denote the MLC leaf movement and cranio-caudal directions, respectively. The largest difference was recorded in the X-direction with the Ø4.0 cm tumour: the 90% isodose line was 4.2 mm narrower with 2 mm SWA than with the 3D-CRT treatment plan (Figure 5). The deviation in the Y-direction with all SWAs and tumour sizes was less than 1 mm. The dynamic IMRT dose distributions of the AAA and the PBC are calculated from the fluence distribution created from actual leaf positions. As a result the dose distributions calculated with AAA (and PBC) with all the SWAs had the same tumour surface doses as the corresponding open field irradiation regardless of the sliding window aperture size since they all generate an uniform radiation distribution.

**Figure 6:**
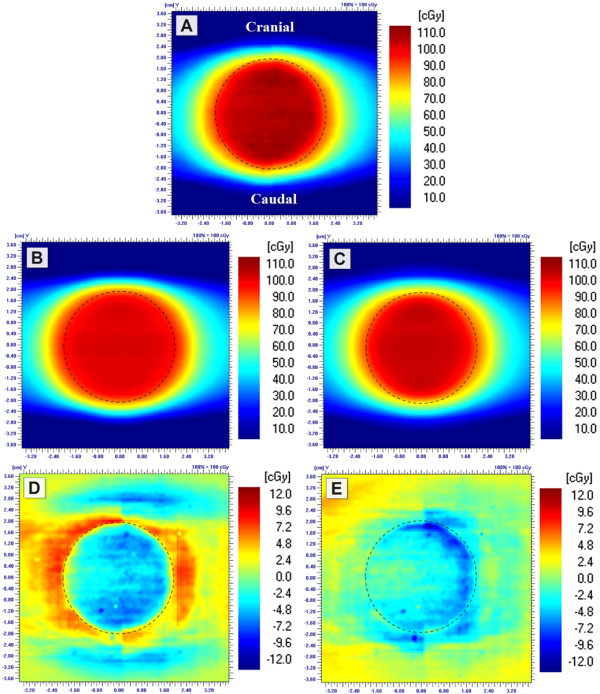
**Measured dose distribution of the IMRT treatment plan of Ø4.0 cm tumour (A) was subtracted from the calculated dose distributions of PBC (B) and AAA (C) (D and E), respectively**. The border line of the tumour is visualized with a dashed line.

As the differences between calculated and measured dose distributions were investigated, the PBC overestimated the peripheral doses, which is evident in Figure 6D. The largest difference was 8.1 mm with an isodose line of 95% while the average difference was 3.8 mm (SD 1.7 mm) with isodose lines of 80-95%. PBC modelled the Y-direction more accurately than the X-direction with an average difference of 2.5 mm (SD 1.4 mm) and 5.0 mm (SD 1.1 mm), respectively. AAA, on the contrary, underestimated the peripheral doses of the tumours with an average error of 1.1 mm (SD 0.6 mm) with the isodose lines of 80-95%. Agreement of the isodose lines was better in the X-direction (0.5 mm, SD 0.5 mm) than in the Y-direction (1.5 mm, SD 0.2 mm). The difference between the measured and calculated (PBC and AAA) isodose line widths of 50% was on average 0.3 mm (SD 0.7 mm). The overall accuracy of PBC and AAA was evaluated with gamma evaluation with normalized central doses. The percentage area of the defined region of interest (ROI: 5 × 5 cm^2^ and 2.5 × 2.5 cm^2^ with large and small tumour sizes, respectively) which failed the criteria of ±3%/±1 mm were on average 47% and 61% with PBC with large and small tumour, respectively. The corresponding average values for the AAA were 13% and 16%, respectively.

## Discussion

The use of IMRT and VMAT techniques is increasing in SLRT and thus the effective field sizes are smaller compared to conventional 3D-CRT. Data evaluating the dosimetric accuracy of modulated techniques applied in SLRT is sparse and possible underdosage of the central or peripheral parts of the tumour may compromise optimal tumour control [26]. The purpose of this study was to evaluate the actual doses of small lung tumours irradiated by intensity-modulated techniques to understand the risk for dose calculation errors in precision RT such as SLRT. The experimental set-up used in this study was chosen such way that the impact of aperture size on the surface doses of simulated tumours could be quantified.

Although our findings are not directly comparable to previous studies it is relevant to relate them to existing data. Sikora *et al.*[18] studied stereotactic lung IMRT dose distributions with radiographic film measurements, Monte Carlo simulations and PBC calculations with tumour sizes of 2.7, 4.2 and 5.0 cm in diameter. The measurements, however, were performed only for a conformal plan consisting of one vertical beam. The central doses were underestimated by PBC approximately by 5% and 6% with 6 MV with tumour sizes of 2.7 cm and 4.2 cm, respectively. In our study, on the contrary, PBC algorithm overestimated the central dose on average 5% for the smaller tumour studied and underestimated the central dose by 1% with the larger tumour. Dobler *et al.*[12] used a conventional treatment plan to irradiate a tumour insert of 2 cm diameter and 4 cm height with 6 MV photons. In their report PBC overestimated the dose up to 5.4% while CC algorithm underestimated the dose by 5.0%. In our study the difference between measured and AAA calculated dose with the 3D-CRT-treatment plan was less than 2%. In a study of Panettieri *et al.*[19] spherical tumours of 2 cm and 5 cm in diameter were studied with conventional treatment fields. Central doses of the tumours were calculated accurately with PBC and CC algorithms but in the target periphery the doses were overestimated up to 10%. In general, PBC tends to overestimate the target dose of small tumours surrounded by lung [27], which is apparent in the smaller tumour of our study (Figure 2). However, with the studied larger tumour (Ø4 cm) PBC algorithm calculated the dose in the central parts of the tumour accurately, but again the peripheral doses were overestimated.

AAA is a more advanced calculation algorithm than the PBC algorithm. Although, there are also limitations in the accuracy of AAA as the divergent scatter of heterogeneities from upper levels is not correctly taken into account and the use of a discrete number of angular sectors might cause smoothening out of the calculated dose distribution near heterogeneous interfaces [28]. With the studied heterogeneous phantoms AAA was able to predict the tumour dose accurately. The difference between the calculated and the measured doses was generally in the range of ±3% except for the very small SWAs and the small tumour, where the difference tended to increase as a function of decreasing SWA (see Figure 2). The main reason for this discrepancy is probably due to the rounded leaf end transmission (dosimetric leaf separation, DLS) and leaf transmission values, which were optimized for conventional treatment plans and for the junction areas of dynamically split IMRT fields. The beam hardening effect becomes also more pronounced by decreasing the SWA because more radiation is penetrating the MLC. Beam hardening effect cannot be modelled in the Eclipse beam configuration since it uses a constant-value model. The values of DLS and leaf transmission used in this study were 0.6 mm and 2.0%, respectively. Chang *et al.*[29] measured the DLS of the HD-MLC to be 0.84 mm for the energy of 6 MV with leaf leakage of 1.0%. From Figure 2 it is evident that very small target volumes are sensitive to the dosimetric settings of the HD-MLC. When the target volume gets larger (in this study Ø4.0 cm) the calculation errors decreases, although the percentage of dose coming from leaf transmission increases. Future studies of the discrepancies between the calculated and measured dose are required to minimize the dose calculation errors in small targets.

The typical values of individual leaf separations with dynamic IMRT treatments are usually between 5 and 15 mm depending on the beam modulation. The average leaf openings with the IMRT treatment plans studied were 12 and 15 mm for the small and large tumour, respectively. With the RA1 treatment plans the corresponding values were 7.9 and 23.7 mm and with the RA2 plans the related values were 7.0 and 13.6 mm. The PBC algorithm underestimated the dose in the centre of the Ø4.0 cm target with the studied IMRT treatment plan by 4% (Figure 2). The main reason for this is that the leaf transmission and DLS values were optimized for the treatment plans calculated with AAA. The optimal values for the PBC calculated treatment plans would be different since the PBC models only the primary photon source and not the scattered photons or electrons from the accelerator head.

The largest deviation of the central doses of the tumours with the IMRT and RapidArc treatment plans was observed with the RA2 treatment plan of the small tumour. AAA underestimated the central dose by 4%, while in the larger tumour the dose was overestimated by 1% (Figure 2). Fog *et al.*[30] observed the same effect in homogeneous phantom with a small target (0.4 cm^3^). In their study the measured doses delivered by RapidArc and HD-MLC were 20% greater than the calculated ones for the small PTV while for the larger PTV there was good agreement between calculated data and measurements. In our study, we also noticed that with greater beam intensity modulation (RA2) the measured dose distribution was less homogenous than the calculated one, which is visible in Figure 3.

The quantity of dose build-up in the vicinity of lung equivalent heterogeneities is proportional to the width of the MLC leaf pairs: the smaller the aperture, the bigger the build-up region at the lung-tumour interface. Indeed, we observed a more than a 2 mm decrease in isodose line width with SWAs of 2 mm and 4 mm to the direction of leaf travel and beam direction (Figure 5). The decrease in tumour peripheral doses was not modelled by the AAA or the PBC. The maximum difference of 4.2 mm in isodose line diameter with the nine field treatment plans was recorded with a SWA of 2 mm and with the Ø4.0 cm tumour, respectively. The highest deviations were recorded with the 85% and 90% isodose lines as can be seen in Figure 5. When the difference was converted to dose difference, the deviation in dose was on average 6%. Surprisingly the difference in isodose line width was smaller with the Ø1.5 cm tumour, which showed a maximum difference of 2.6 mm. With a SWA larger than 6 mm the maximum difference between isodose line radius of open and modulated treatment plan was decreased down to 1 mm. The main reason for the surface dose difference between the open and modulated fields is probably due to the increased rebuild-up and rebuild-down effects at the mimicked tumour and lung interfaces with the small apertures.

The dosimetric inaccuracy of a single EBT2 film measurement has been reported to be 3.8% in the dose range of 0–4 Gy [25]. We repeated the measurements three times to increase the overall accuracy of the used EBT2 dosimeter. The individual film measurements were presumed to be normally distributed and the SD of the film measurements was used to estimate the measurement inaccuracy, which was on average 1.6%.

Our study is impeded by few limitations. We neglected the movement of the tumour caused by breathing and the setup of the study is thus best comparable to treating patients with active breath hold techniques or having tumours with a very limited movement. Alternatively respiration-correlated 4D-CT can be used to visualize tumour motion and treatment plan optimization can be realised with average CT or maximum intensity projection (MIP) CT [31]. It has been shown that calculation algorithms overestimate the peripheral doses of moving tumours and the underdosing effect is not noticed in the central parts of the targets [19]. Accordingly, we can conclude that if the studied tumours were not stationary, the peripheral doses were likely to decrease even more than reported in this study.

The dose calculation accuracy in the patient geometry does not only depend on the used calculation algorithm but also on patient anatomy, used energy, and effective field size. This study was based on the assumption that the entire tumour is surrounded by lung. However, tumours might be situated close to the thoracic wall or even attached to it, which would result in a decrease of dose build-up effects.

## Conclusions

Narrow field sizes produced by HD-MLC were studied to find the possible limitations of using small dynamic apertures in SLRT. The surface doses of the mimicked lung tumours decreased with decreasing the sliding window aperture. The largest deviation between measured and calculated isodose lines was 4.2 mm, which was recorded with a SWA of 2 mm and with the larger tumour size studied. With a larger than 6 mm SWA the difference in the isodose line radius between the open and modulated field was less than 1 mm. AAA was able to calculate the peripheral doses of the tumours accurately with an average error of 1.1 mm as the measured and calculated isodose lines of 80-95% were compared. The PBC algorithm failed to model the peripheral doses of the small lung tumours correctly and thus should be used with caution in the treatments of SLRT.

## Competing interests

The authors have no financial disclosures or conflicts of interest to report.

## Authors’ contributions

JS, SS made substantial contributions to the conception and to data acquisition and analysis and drafting the manuscript. JK participated in the study design and coordination and helped to draft the manuscript. PM, HM have been involved in designing the study, drafting the manuscript and critically revising the study. All authors read and approved the final manuscript.
